# *RcAP1*, a Homolog of *APETALA1*, is Associated with Flower Bud Differentiation and Floral Organ Morphogenesis in *Rosa chinensis*

**DOI:** 10.3390/ijms20143557

**Published:** 2019-07-20

**Authors:** Yu Han, Aoying Tang, Jiayao Yu, Tangren Cheng, Jia Wang, Weiru Yang, Huitang Pan, Qixiang Zhang

**Affiliations:** 1 Beijing Key Laboratory of Ornamental Plants Germplasm Innovation & Molecular Breeding, National Engineering Research Center for Floriculture, Beijing Laboratory of Urban and Rural Ecological Environment, School of Landscape Architecture, Beijing Forestry University, Beijing 100083, China; 2 Key Laboratory of Genetics and Breeding in Forest Trees and Ornamental Plants of Ministry of Education, School of Landscape Architecture, Beijing Forestry University, Beijing 100083, China; 3 Beijing Advanced Innovation Center for Tree Breeding by Molecular Design, Beijing Forestry University, Beijing 100083, China

**Keywords:** *APETALA1*, *Rosa chinensis*, sepal, flower bud differentiation, floral organ morphogenesis

## Abstract

*Rosa chinensis* is one of the most popular flower plants worldwide. The recurrent flowering trait greatly enhances the ornamental value of roses, and is the result of the constant formation of new flower buds. Flower bud differentiation has always been a major topic of interest among researchers. The *APETALA1* (*AP1*) MADS-box (Mcm1, Agamous, Deficiens and SRF) transcription factor-encoding gene is important for the formation of the floral meristem and floral organs. However, research on the rose *AP1* gene has been limited. Thus, we isolated *AP1* from *Rosa chinensis* ‘Old Blush’. An expression analysis revealed that *RcAP1* was not expressed before the floral primordia formation stage in flower buds. The overexpression of *RcAP1* in *Arabidopsis thaliana* resulted in an early-flowering phenotype. Additionally, the virus-induced down-regulation of *RcAP1* expression delayed flowering in ‘Old Blush’. Moreover, *RcAP1* was specifically expressed in the sepals of floral organs, while its expression was down-regulated in abnormal sepals and leaf-like organs. These observations suggest that *RcAP1* may contribute to rose bud differentiation as well as floral organ morphogenesis, especially the sepals. These results may help for further characterization of the regulatory mechanisms of the recurrent flowering trait in rose.

## 1. Introduction

Roses, which are among the most popular flowers worldwide, have been cultivated in China since 3000 B.C. There are currently more than 35,000 rose cultivars. The genus *Rosa* includes more than 130 recognized species [1]. Rose plants are suitable for studying flower development in woody plant species because of the observed differences in flower morphology among roses and the fact that their continuous flowering trait makes it relatively easy to collect flower tissue. Currently, rose has become one of the most widely cultivated ornamental plants and it also has a huge demand in the perfume and cosmetics industry. 

Flowering is a very complex biological phenomenon, and the meristem of flower organs is affected by external and endogenous factors during the process from vegetative growth to reproductive development [2,3,4]. MADS-box proteins are important transcription factors in plants that play important roles in flower development and control the generation of flower organ, as is described in the ABCDE model [5,6,7]. In the ABCDE model, *APETALA1*(*AP1*) and *APETALA2*(*AP2*) belong to A-functional genes and these genes are responsible for the formation of sepals in the first whorl [8,9]. B-functional genes includes *APETALA3* (*AP3*) and *PISTILLATA* (*PI*), while *AGAMOUS* (*AG*) is a C-functional gene [10]. Theissen et al. (2001) put forward the tetrameric model of floral development in the MADS-box gene. This model represents the five types of MADS-box proteins (A, B, C, D, and E) that regulate the development of different flower organs by forming tetramers. Studies in *Arabidopsis thaliana*, *Petunia hybrida*, and *Antirrhinum majus* have revealed that MADS-box transcription factors regulate floral organ formation [11,12]. In rose plants, MADS-box orthologs involved in floral organ formation have also been cloned and partially functionally verified, such as *RhAP3*, *RhPI*, and *RhAG* [13,14]. 

*AP1* is a subfamily of the MADS-box gene that has been identified as a floral meristem gene, which induces floral development in many species [15,16]. *AP1* and *LEAFY* (*LFY*) are two floral meristem identity genes that both regulate the specification and formation of floral meristems on the flank of the shoot apical meristem [17,18]. *AP1* and *LFY* act together in the early stage of flowering. After the transformation, the expression of *AP1* seems to be only indirectly affected by *LFY* [19]. Additionally, it has been certified that *AP1*, *FRUITFULL* (*FUL*), and *CAULIFLOWER* (*CAL*) genes control floral meristem characteristics and inflorescence structure, meanwhile *AP1* and *CAL* genes are involved in the development of sepal and petal primordial cells [20]. 

Of the four whorls of floral organs, *AP1* is specifically expressed in the first and second whorls of floral buds [21]. In some ornamental plants, *AP1* orthologs have been isolated, such as chrysanthemum [22], lily [23], poppy [24], camellia [25], and gentian [26]. Several studies imply that *AP1* is critical for floral formation. The effects of AP1 in some species with diverse flowering characteristics have also been described, such as *Jatropha curcas (JcAP1)* and *Salix discolor* (*SAP1*), and the overexpression of these genes in *A. thaliana* may lead to different phenotypes [22,23,24,25,26,27,28]. Although *AP1* genes are conserved to some degree, they may be associated with a variety of functions. Additionally, *AP1* and its orthologs often affect the following two distinct but linked aspects of flowering: Flower meristem identity and sepal specification [7,29,30,31].

In rose, continuous flowering has been widely considered as one of the ornamental characteristics, which has greatly increased the viewing period of modern rose flowers. The essence of recurrent flowering is continuous flower bud differentiation [32]. Thus, the function analysis of the *AP1* gene in *R. chinensis* is necessary. In this study, we isolated a homolog of APETALA, *RcAP1*, from *R. chinensis* ‘Old Blush’, and analyzed the promoter and encoded protein sequences, as well as the evolutionary relationships with genes from other species. Moreover, *RcAP1* was characterized by investigating its expression patterns and examining its biological effects in transgenic *A. thaliana* plants. We also silenced *RcAP1* via a virus-induced gene silencing (VIGS) procedure. The data presented herein may enrich our understanding of the flower formation mechanism in rose.

## 2. Results

### 2.1. Bioinformatics Analysis of APETALA1

**Figure 1:**
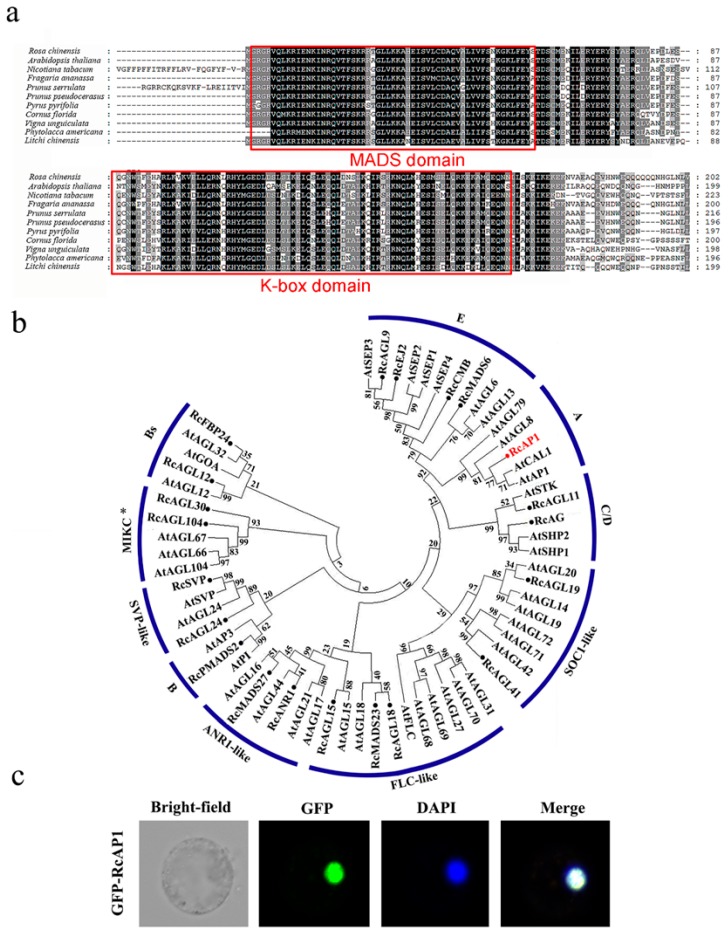
Bioinformatic analysis of *RcAP1* (**a**) Multiple sequence alignment of deduced amino acid sequences encoded by *RcAP1* and other *AP1* genes from diverse species. Identical conserved residues are indicated in black, while partially conserved residues are presented in gray. The dotted lines indicate gaps to maximize the alignment. The conserved MADS and K-box domains are framed in red. (**b**) Phylogenetic analysis of *Rosa chinensis* and *A. thaliana* type II MADS-box proteins. A total of 21 *R. chinensis* type II MADS-box proteins and 41 *A. thaliana* type II MADS-box proteins were included in a phylogenetic tree constructed according to a neighbor-joining method. The *RcAP1* investigated in this study is highlighted in red. The *R. chinensis* type II MADS-box proteins are marked with solid circles. (**c**) Subcellular localization of *RcAP1*. The *RcAP1*-GFP (Green Fluorescent Protein) fusion protein localized to the nucleus of maize protoplasts.

We isolated an *RcAP1* gene encoding a polypeptide with 247 aa residues. A multiple sequence alignment (Figure 1a) revealed that *RcAP1* was most similar (i.e., highest sequence identity) to sequences from Rosaceae plants, but was also highly similar to genes from other species (>75%). They all have a MADS MEF2-like sequence at the N-terminus and a highly conserved K-box domain, and belong to the type II MADS-box proteins. Using the phylogenetic analysis with *A. thaliana*, 21 *R. chinensis* type II MADS-box proteins were classified into 10 groups, including the A, B, C, D, E, *FLOWERING LOCUS F (FLC)*-like, *SUPPRESSOR OF OVEREXPRESSION OF CO 1 (SOC1)*-like, *ARABIDOPSIS NITRATE REGULATED 1 (ANR1)*-like, and S*HORT VEGETATIVE PHASE (SVP)*-like functional groups in subfamilies MIKCC and MIKC*. The *RcAP1* was classified as an A-class gene. The evolutionary relationships about *AP1* in *R. chinensis* and other species are shown in Figure S1 and *RcAP1* may be an ortholog of *APETALA1*. The encoded protein was localized in the nucleus of maize protoplasts (Figure 1c) and the nucleus of *Nicotiana benthamiana* leaves (Figure S2) based on an analysis of *RcAP1*-GFP fluorescence.

We isolated the *RcAP1* promoter, which consisted of a 2003 bp DNA sequence upstream of the start codon. Additionally, online programs were used to predict the cis-acting elements of the *RcAP1* promoter. On the ‘+’ strand, the CCGTCC-box (nucleotides −429 to −423) was associated with meristem-specific activation, while the EM2 sequence (nucleotides −1323 to −1314) was related to floral meristem determinacy. The RY site (nucleotides −1177 to −1169) was involved in the induction of flowering. Meanwhile, the ‘-’ strand included the DREB2A (nucleotides −1079 to −1087) and HSE-1 (nucleotides −713 to −722) sequences, which were related to cold stress and heat stress responses, respectively. Details are provided in Table S1. 

### 2.2. RcAP1 is Highly Expressed during the Bud Appearance Stage and is Mainly Expressed in Sepals

**Figure 2:**
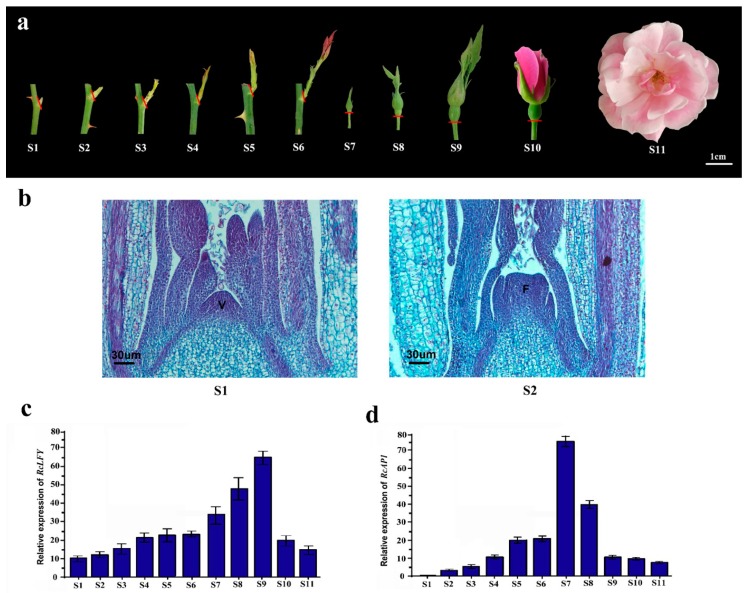
Spatiotemporal pattern of *RcAP1* expression in ‘Old Blush’ plants. (a) Photographs of the 11 flower development stages of ‘Old Blush’ plants (S1–S11). S1: vegetative cone and leaf primordia formed; S2: floral meristem formed; S3: sepal primordia formed; S4: petal primordia formed; S5: stamen primordia formed; S6: pistil primordia formed; S7: small flower buds formed (approximately 2 mm diameter); S8: moderate-sized flower buds formed (approximately 4 mm diameter); S9: flower bud differentiation was completed, but sepals did not crack; S10: flowers started to open; and S11: flowers were completely open. Red lines represent the cut marks of the rose parts used for RNA isolation. Scale bar, 1 cm. (**b**) Paraffin sections of ‘Old Blush’ buds stained with toluidine blue in S1 and S2. V, vegetative cone; F, floral meristem. Scale bar, 30 µm. (**c**) Results of a qRT-PCR analysis of *RcLFY* transcript levels in the 11 rose flower development stages. (**d**) Results of a qRT-PCR analysis of *RcAP1* transcript levels in the 11 rose flower development stages. Error bars represent the standard deviation.

The rose flower development process was divided into 11 stages (S1–S11) (Figure 2a). To confirm the transition from vegetative to reproductive growth, we prepared paraffin sections at S1 and S2 (Figure 2b). The floral meristem emerged during S2. To analyze the spatiotemporal *RcAP1* expression pattern, we used *RcLFY* (‘Old Blush’, GenBank ID MN119279) as a floral marker. In *A. thaliana*, *LFY* is a meristem identity gene, and the encoded protein activates *AP1* expression [33,34]. During all 11 flower development stages, the expression of *RcLFY* was consistent with that of *RoLFY* in *R. hybrida* ‘Little White Pet’ [35] (Figure 2c). Our data indicated that *RcAP1* was not expressed during S1 (Figure 2d), in which the floral meristem was absent. Additionally, *RcLFY* was expressed at S1, which is earlier than *RcAP1* during bud development. In contrast, *RcAP1* expression increased and peaked in S7, before decreasing to half in S8 and remaining low during S9 to S11.

**Figure 3:**
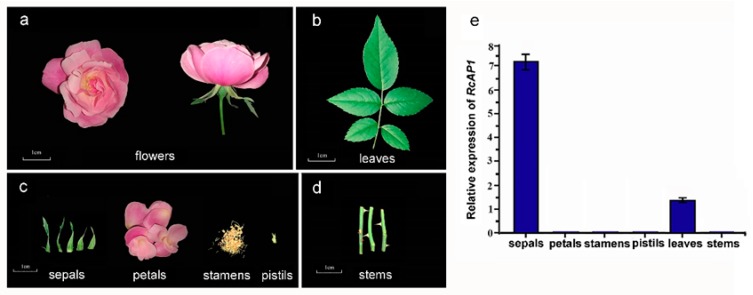
Expression of *RcAP1* in different ‘Old Blush’ organs. (**a**) Front and side views of fully-bloomed flowers. (**b**) Compound leaves. (**c**) Floral organs, including sepals, petals, stamens, and pistils. (**d**) Stems. Scale bar, 1 cm. (**e**) Quantitative analysis of *RcAP1* expression in different ‘Old Blush’ organs. Error bars represent the standard deviation.

**Figure 4:**
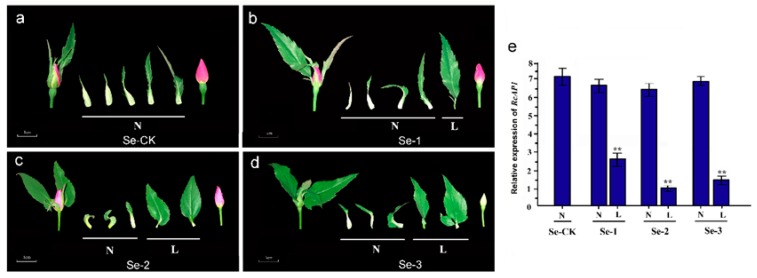
Expression of *RcAP1* in normal and leaf-like ‘Old Blush’ sepals. (**a**) Normal ‘Old Blush’ sepals served as controls (Se-CK). N, normal sepals. (**b**) Normal and leaf-like sepals (Se-1). L, leaf-like sepals. (**c**) Normal and leaf-like sepals (Se-2). (**d**) Normal and leaf-like sepals (Se-3). Scale bar, 1 cm. (**e**) Quantitative analysis of *RcAP1* expression in normal and leaf-like ‘Old Blush’ sepals. ** indicates significant differences (*p* < 0.01) according to Dunnett’s test. Error bars represent the standard deviation.

The tissue-specificity of *RcAP1* expression was also examined. A quantitative real-time polymerase chain reaction (qRT-PCR) analysis indicated that *RcAP1* was highly expressed in sepals, expressed at relatively low levels in leaves, and not expressed in petals, stamens, pistils, and stems (Figure 3). There were significant differences between the expression levels in sepals and leaves. During the cultivation of rose, we observed that under heat stress, sepals could develop into more leaf-like organs. Since *AP1* regulates sepal development in many species, we investigated whether *RcAP1* expression was different in sepals under heat stress. A relative quantitative gene expression analysis of sepals exposed to high-temperature stress (32 °C (light): 22 °C (dark), two weeks) or normal conditions was completed (Figure 4). Under high-temperature stress conditions, there were no significant differences in *RcAP1* expression in the morphologically normal sepals (Se-1, Se-2, and Se-3). Additionally, the *RcAP1* expression levels were similar to that of Se-CK (i.e., ‘Old Blush’ sepals under normal conditions) (Figure 4e). Among the morphologically altered sepals, the *RcAP1* expression levels decreased as the similarity between the sepal and leaf shapes increased. The shape of the Se-2 abnormal sepals with obvious main veins was the most similar to that of a normal individual leaflet.

**Figure 5:**
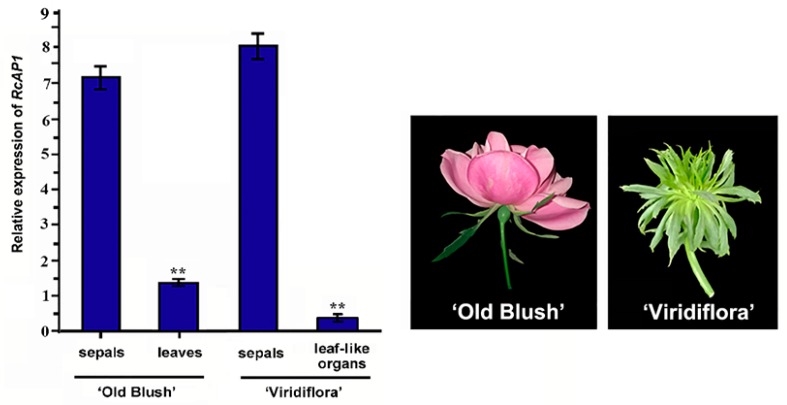
Quantitative analysis of *RcAP1* expression in ‘Old Blush’ and ‘Viridiflora’ plants. Asterisks indicate significant differences (*p* < 0.01) according to Dunnett’s test. Error bars represent the standard deviation.

The *RcAP1* expression level was lower in leaf-like sepals than in normal sepals (Figure 4). Of the various ‘Old Blush’ organs, *RcAP1* was expressed in the sepals and leaves, but not in the other floral organs. *Rosa chinensis* ‘Viridiflora’ is a natural variant in which petals, stamens, and pistils appear as leaf-like organs. Because of the specific *RcAP1* expression patterns in normal and abnormal sepals, we examined other plant tissues to test whether *RcAP1* expression is related to the floral organ morphology. A qRT-PCR analysis of ‘Old Blush’ leaves and sepals as well as ‘Viridiflora’ sepals and leaf-like organs revealed that *RcAP1* expression levels were lowest in the leaf-like organs (Figure 5), and were also relatively low in ‘Old Blush’ leaves. Meanwhile, *RcAP1* was highly expressed in the sepals of the two rose cultivars.

### 2.3. Overexpression of RcAP1 in Arabidopsis Thaliana Induces Early Flowering

**Figure 6:**
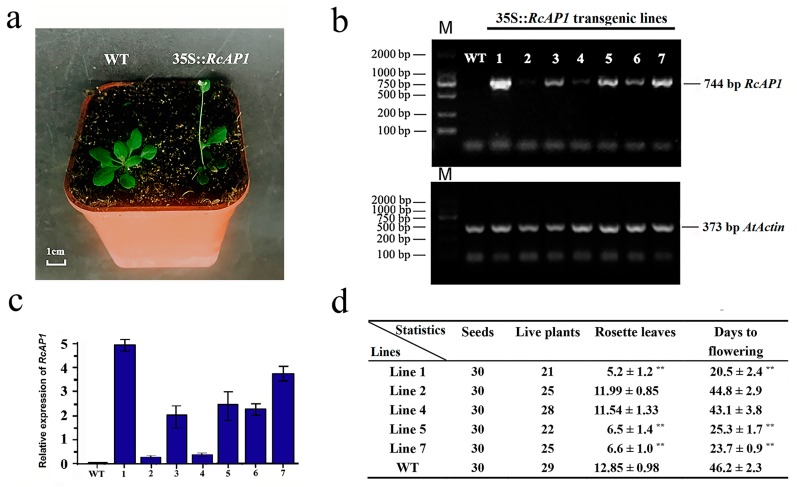
Overexpression of *RcAP1* induces early flowering in transgenic *A. thaliana* plants. (**a**) Representative 35S::*RcAP1* transgenic plant exhibiting early flowering under long-day conditions. Bar, 1 cm. (**b**) Results of an RT-PCR analysis of *RcAP1* expression in transgenic *A. thaliana* plants. M, Marker DL 2000; WT, Wild-type plants; 1–7, independent 35s::*RcAP1* transgenic lines. AtActin, which was used as an internal control, was amplified with primers *AtActin*-F and *AtActin*-R. (**c**) Results of a qRT-PCR analysis of *RcAP1* transcripts in transgenic *A. thaliana* lines. Wild-type plants served as controls. (**d**) Number of seeds, live plants, and rosette leaves, as well as days to flowering in WT and transgenic *A. thaliana* lines. Values are presented as the mean ± standard deviation of three biological replicates. ** indicates significant differences (*p* < 0.01) according to Dunnett’s test. Error bars represent the standard deviation.

We analyzed the plant phenotype when *RcAP1* was overexpressed in wild-type (WT) *A. thaliana*. Seven independent T1 generation transgenic lines were detected based on hygromycin resistance, and subsequently confirmed with RT-PCR and qRT-PCR analyses (Figure 6b,c). Five representative transgenic lines were chosen for further analysis. According to the qRT-PCR data, line 2 and line 4 represent the low level overexpression, line 5 and line 7 represent the middle level overexpression, and line 1 represents the highest level overexpression of *RcAP1* in *A. thaliana*. Lines 1, 2, 4, 5, and 7 (L1, L2, L4, L5, and L7) were screened using the selection method described above until T3 generation transgenic seeds were obtained. In total, 30 seeds were sown for five independent homozygous transgenic lines (L1, L2, L4, L5, and L7) and the WT control. The resulting plants were cultured in an incubator under long-day conditions (16-h light/8-h dark). Three of the transgenic lines (L1, L5, and L7) exhibited varying degrees of early flowering (Figure 6a and Figure S4b). The rosette leaves were counted and the days to flowering were determined (Figure 6d). Of the transgenic lines, L1 exhibited the earliest flowering (day 18 after sowing), while L5 exhibited the latest flowering (day 27 after sowing). The T3 plants of L1, L5, and L7 bloomed earlier than the WT plants, which started to bloom on day 44 after sowing. Meanwhile, L2 and L4, which exhibited low *RcAP1* expression levels with no obvious early flowering, served as the negative controls for L1, L5, and L7. The early flowering transgenic plants produced almost half as many rosette leaves per plant as the WT plants. This was likely because *RcAP1* expression induced flowering when there were relatively few leaves.

### 2.4. Knockdown of RcAP1 Expression in ‘Old Blush’ Inhibits Flower Bud Formation

**Figure 7:**
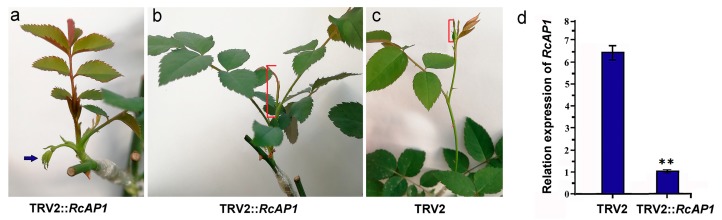
Knockdown of *RcAP1* in ‘Old Blush’ plants. (**a**) Scions of TRV2::*RcAP1* after 30 days. The blue solid arrow indicates small incompletely developed compound leaves at the bottom of TRV2::*RcAP1* scions. (**b**) Scions of TRV2::*RcAP1* after 50 days. (**c**) Scions of TRV2 after 50 days. The red marks indicate the rose parts used for RNA isolation. (**d**) Quantitative analysis of *RcAP1* expression in TRV2::*RcAP1* and TRV2 scions. Error bars represent the standard deviation. ** indicates significant differences (*p* < 0.01) according to Dunnett’s test. Error bars represent the standard deviation.

The VIGS method was used to knock down the *AP1* gene in ‘Old Blush’ plants. Approximately 30 days after injections, small compound leaves that were incompletely developed appeared at the bottom of the TRV2::*RcAP1* scions (Figure 7a). After 50 days, the compound leaves at the bottom of shoots in the TRV2::*RcAP1* scions had expanded but had no visible flower buds (Figure 7b). While on the TRV2 control scions, flower buds had formed and were about to open (Figure 7c). Total RNA was extracted from the flower buds of the 50-day-old TRV2 control scions, which may already have been at S8 or S9, as well as from the 2-cm shoot tips (including unexpanded leaves) of the TRV2::*RcAP1* scions. A qRT-PCR analysis revealed significant differences between the TRV2 and TRV2::*RcAP1* plants regarding the relative *RcAP1* expression levels (Figure 7d). The TRV2::*RcAP1* plants exhibited low relative *RcAP1* expression levels. The *RcSOC1* and *RcFUL* genes, which are likely downstream targets of *RcAP1*, were cloned. In *A. thaliana*, *SOC1* is inhibited by *APETALA1* in emerging floral meristems [36], and *FUL* is also negatively regulated by *APETALA1* [37]. We analyzed the expression of *RcSOC1* and *RcFUL* in 50-day-old TRV2::*RcAP1* plants (Figure S5). As important genes involved in flower bud differentiation, the up-regulated expression of *RcSOC1* and *RcFUL* indicated that they may be regulated by *RcAP1*. Thus, the down-regulation of *AP1* expression may inhibit the formation of flower buds and delay flowering. However, there were no observable differences between the TRV2 control and TRV2::*RcAP1* plants regarding floral morphology.

## 3. Discussion

Sequence analyses and multiple sequence alignments confirmed that *RcAP1* ORF cloned from *R. chinensis* ‘Old Blush’ is similar to other *AP1* genes from different species. These genes carry conserved sequences encoding the MADS-box and K-box domains, which are conserved domains of the MADS-box family. The presence of the MADS-box domain implies that the protein can bind to DNA and function as a transcription factor [38]. The localization of the GFP-RcAP1 fusion protein in the nucleus also suggests that *RcAP1* may exhibit transcription factor activities. The K-box domain, which is a characteristic sequence of transcription factors, is responsible for protein interactions [39]. The phylogenetic tree analysis revealed that *RcAP1* is highly homologous to the *AP1* genes of other plant species, with the highest similarity to sequences from Rosaceae plants.

Despite the sequence conservation of *RcAP1* as discussed above, some of our results also indicate other potential functions of *RcAP1* in flower development. Previous studies revealed that *AP1* promotes the transition from vegetative growth to reproductive growth. Here, we observed that *RcAP1* was not expressed in buds at S1, during which there are no floral primordia. Additionally, the *RcAP1* expression level started to increase at S2. The expression of *AP1* in various tissues and floral organs has been reported in some Rosaceae species. Randoux et al. (2012) used qPCR to analyze gene transcripts’ accumulation in response to gibberellic acid A3 (GA3) treatment. They detected the accumulation of *RoAP1* (*Rosa × wichurana*) transcripts in the absence of treatment [40]. In an earlier investigation, the results of a northern blot revealed that *Eriobotrya japonica AP1* was highly expressed in sepals, lowly expressed in petals and stamens, and not expressed in leaves [41]. Furthermore, a qRT-PCR assay determined that *Prunus avium AP1* was expressed mainly in petals, sepals, and styles, with lower expression levels in carpels, stamens, and some vegetative tissues (young stems and leaves) [42]. *Pyrus pyrifolia AP1* transcripts were mainly detected in sepals, with a lower abundance in petals and stamens, and a lack of transcripts in pistils, leaves, and roots [43]. Yan et al. (2016) used the qRT-PCR assay and found that *RcAP1* is expressed in the first and second flower whorls in *R. chinensis* cv. Viridiflora, as well as in the first flower whorl in ‘Old Blush’. However, *RcAP1* was not expressed in leaves. Yan’s study mainly focused on flower organ identity determination and development [44]. In our study, we observed that the transcripts of *RcAP1* only accumulated in sepals among four flower wholes of a normal flower in ‘Old Blush’. However, in abnormal phenotypes, including the ‘Old Blush’ leaf-like sepals resulting from an exposure to high temperatures and the leaf-like floral parts in the natural rose variant ‘Viridiflora’, *RcAP1* has a lower expression than in normal sepals. We also detected a low expression of *RcAP1* in rose leaves. Similar data were observed in *R. hybrida* [45]. The floral organ-specific distribution of *AP1* was different between many flowering plants. These differences in *RcAP1* expression patterns imply that *RcAP1* may influence floral organ morphology in addition to affecting floral meristem formation.

In *A. thaliana*, *AP1* overexpression results in significantly earlier flowering [15]. So far, *AP1* orthologs have also been isolated in a wide range of ornamental plants, such as *PtAP1* (*Poncirus trifoliata*) [46], *PaAP1* (*Pyrus pyrifolia*) [43], *DoAP1* (*Orchid Dendrobium*) [47], *JcAP1* (*Jatropha curcas*) [28], and *SAP1* (*Salix discolor*) [27]. Moreover, we found the ectopic expression of *RcAP1* in *A. thaliana* also induces early flowering. In addition to promoting flowering, *AP1* from different plant species exhibit functional diversity. The expression of *Poncirus trifoliata AP1* in *A. thaliana* induces the production of abnormal leaf inflorescences, leading to a curled rosette and cauline leaves [46]. In *A. thaliana* transgenic lines expressing *SAP1*, the terminal flowers have six petals or three pistils with more petals [27]. Thus, although *AP1* functions are partially conserved in diverse species, there are also some differences.

The early flowering phenotype observed in *RcAP1*-overexpressing transgenic *A. thaliana* plants is evidence that *RcAP1* may be involved in flower bud differentiation, but due to its ectopic expression of *RcAP1*, this evidence was not enough. A novel VIGS method was presented and can be used to analyze gene function in rose (i.e., graft-accelerated VIGS), and the silencing phenotypes can be observed within 5 weeks after *Agrobacterium*-infiltration [48]. Using this method, we observed that VIGS-mediated silencing of *RcAP1* in ‘Old Blush’ plants delayed the formation of flower buds and flowering. However, the flowering time of our treated and control plants increased by more than 5 weeks. We speculate that this was because of differences between our study and the reference study regarding the scion status, environmental factors, or manipulation methods [48]. Unlike the controls, the flower buds of TRV2::*RcAP1* plants were not visible on day 50. However, we did not detect differences between the TRV2 control and TRV2::*RcAP1* plants regarding the floral phenotype, possibly because of the temporary nature of the VIGS-mediated silencing or the redundancy in the functions of MADS-box genes, such as *CAL* [49]. Up-regulated *RcSOC1* and *RcFUL* expression levels were detected in TRV2::*RcAP1* plants, which represent additional evidence that the down-regulated expression of *RcAP1* affects rose bud differentiation. Since about 25 years ago, the involvement of *AP1* in the control of flowering has been well-known [15,29], and research on the functions of *AP1* has continued. Our results have shown that an *AP1* from rose controls flowering, which is relevant for future investigations of the regulation of flowering in rose varieties. However, in addition to the expression characteristics in flower meristems and the function of regulating flowering, the specific expression of *RcAP1* in sepals and its ability to participate in the regulation of sepal morphology were also worthy of attention, because the pattern of rose flowers determines their ornamental value. The temporal and spatial expression of *RcAP1* may be related to different biological traits. What is the regulatory mechanism of the expression characteristics of *RcAP1*? The interaction proteins and downstream target genes of *RcAP1*, and the transcription factors that regulate *RcAP1* expression can be further studied to reveal the conservation and diversity of *RcAP1* gene functions.

We identified an A-class rose *AP1* gene. Ectopic expression experiments involving *A. thaliana* and knockdown experiments in *R. chinensis* proved that *RcAP1* may be involved in the formation and differentiation of rose buds. Furthermore, *RcAP1* expression patterns in floral organs, deformed sepals, and leaf-like organs indicated that *RcAP1* may influence rose floral organ morphogenesis. The data presented herein may provide new insights into the regulatory mechanisms of rose floral organs and may be useful for comprehensive analyses of flower opening.

## 4. Materials and Methods

### 4.1. Plant Materials and Growth Conditions

*R. chinensis* ‘Old Blush’ plants were grown in flower pots in a greenhouse at Beijing Forestry University (Beijing, China) under a 24 °C day/14 °C night temperature regime and a 16-h-light/8-h-dark photoperiod. The relative humidity was set at 40% to 60% and photoperiods were among 30,000 to 60,000 lx. *R. chinensis* ‘Viridiflora’ plants, an ancient naturally occurring mutant of ‘Old Blush’ in which petals, stamens, and pistils are converted to leaf-like organs [44], were cultivated in a plant growth chamber at the same condition for collecting leaf-like organs. The flower development process of ‘Old Blush’ plants was divided into the following 11 stages according to a previous study: S1: Vegetative cone and leaf primordia formed; S2: Floral meristem formed; S3: Sepal primordia formed; S4: Petal primordia formed; S5: Stamen primordia formed; S6: Pistil primordia formed; S7: Small buds formed (approximately 2 mm diameter); S8: Moderate-sized buds formed (approximately 4 mm diameter); S9: Bud differentiation was completed, but sepals did not crack; S10: Flowers started to open; and S11: Flowers were completely open. For each stage, 10 shoots, flower buds, or flowers were collected and combined, after which they were frozen in liquid nitrogen. From S1 to S6, the shoots were cut off at the part connected to the stem and immediately put into the liquid nitrogen. From S7 to S11, the whole flower buds or flowers were cut and collected. Total RNA was extracted from the collected samples for a qRT-PCR validation. Total RNA was extracted from ‘Old Blush’ stems, leaves, petals, normal and abnormal sepals, stamens, and pistils as well as ‘Viridiflora’ leaf-like organs harvested during S11. Wild-type (ecotype Columbia) and transgenic *A. thaliana* plants were grown in a light incubator at 22 °C under long-day conditions (16-h light/8-h dark).

### 4.2. Paraffin Sections and Staining

To observe the structures of buds in S1 and S2 of ‘Old Blush’, the buds were embedded in paraffin and dyed with toluidine blue (Sigma-Aldrich, St Louis, MO, USA), according to previously published approaches [50].

### 4.3. RNA and DNA Extraction

Total RNA was extracted from the collected samples using the EASYspin plant RNA kit (Aidlab Biotech, Beijing, China), while DNA was extracted using the Plant Genomic DNA kit (Omega Bio-tek Inc., Norcross, GA, USA).

### 4.4. ORF and Promoter Cloning

Total RNA was extracted from ‘Old Blush’ flower buds. First-strand cDNA was synthesized using the TIANScript RT Kit (Tiangen Biotech, Beijing, China). The sequences of the conserved regions of *AP1* homologs from *A. thaliana*, strawberry, pear, citrus, and other species were downloaded from the NCBI (National Center for Biotechnology Information) database. These sequences were used to manually design two degenerate primers (*RcAP1*-F and *RcAP1*-R) with Primer Premier 5.0 to amplify the middle fragment of *RcAP1*. The PCR products were purified using the TIANgel Midi Purification Kit (Tiangen) and cloned into the TOPO vector with the Zero Background Omni TOPO Cloning Kit (Taihegene, Beijing, China). The vector was then inserted into *Escherichia coli* DH5α cells. Eight positive colonies were sequenced (Taihegene), after which the Clontech SMARTer^®^ RACE 5′/3′ Kit (Takara Bio Inc., Shiga, Japan) was used to obtain the full-length *RcAP1* cDNA. Additional primers (3′GSP and 5′GSP) were designed according to the RACE (Rapid-Amplification of cDNA Ends) primer design principle, while the universal primer (UPM) in the kit served as a paired primer. The two sequences obtained by RACE were assembled (DNAman 8.0) and then analyzed using the NCBI ORF Finder to locate the coding sequence. End-to-end primers (*RcAP1*-full-F and *RcAP1*-full-R) were designed and used to amplify the *RcAP1* ORF by PCR.

According to the manufacturer’s instructions for the Genome Walking Kit (Takara), we designed three primers (*RcAP1*-pro-SP1, *RcAP1*-pro-SP2, and *RcAP1*-pro-SP3) to amplify *RcAP1* promoter fragments by PCR, with DNA isolated from flower buds as the template. After cloning and sequencing, the expected promoter sequence was obtained. The PlantCARE (http://bioinformatics.psb.ugent.be/webtools/plantcare/html/) [51] and SoftBerry (http://linux1.softberry.com/berry.phtml?topic=nsitep&group=programs&subgroup=promoter) [52] online programs were used to analyze the cis-acting elements of the *RcAP1* promoter. Detailed information about the *RcAP1* promoter (GenBank ID MH556915) and coding sequence (GenBank ID MG779281) was deposited in the NCBI database (http://www.ncbi.nlm.nih.gov/genbank/). According to previous studies and using the homologous gene alignment method, we cloned two possible downstream targets of *RcAP1*, named *RcSOC1* (GenBank ID MN119277) and *RcFUL* (GenBank ID MN119278), and qPCR primers were designed. Details regarding all primers are provided in Table S2.

### 4.5. Sequence and Phylogenetic Analyses

The *RcAP1* aa sequence was deduced based on the nucleotide sequence. On the basis of BLAST (Basic Local Alignment Search Tool) searches of the NCBI GenBank database, we selected 10 representative *AP1* MADS-box genes from typical model plants as well as Rosaceae and other species for a multiple sequence alignment using ClustalX. The GenBank IDs of the AP1 aa sequences included in multiple sequence alignment analyses were as follows: Z16421.1 *Arabidopsis thaliana*; AF009127.1 *Nicotiana tabacum*; JN788262.1 *Fragaria ananassa*; GQ267074.1 *Prunus serrulata*; KM243377.1 *Prunus pseudocerasus*; EF423916.1 *Pyrus pyrifolia*; KT965645.1 *Cornus florida*; AB588744.1 *Vigna unguiculata*; AY306160.1 *Phytolacca americana*; and JN214349.1 *Litchi chinensis*.

The aa sequences encoded by type II MADS-box genes were obtained from the *R. chinensis* ‘Old Blush’ genome database (https://lipm-browsers.toulouse.inra.fr/pub/RchiOBHm-V2/) and the PlantTFDB *A. thaliana* database (http://planttfdb.cbi.pku.edu.cn/). Using BLAST algorithm-based searches of the NCBI GenBank databases, we selected 18 representative *AP1* MADS-box genes from typical model plants, Rosaceae plants, and other species for the construction of a phylogenetic tree. The ClustalX method was used for performing the multiple sequence alignment of the 19 genes. The neighbor-joining algorithm-based phylogenetic tree was generated using the Poisson model, with uniform rates and 1000 bootstrap replicates, by the MEGA 7.0 software. The conserved domain of each gene was used as a query in the NCBI conserved domain database, and iTOL (http://itol.embl.de/) was used to complement the conserved domain diagram.

### 4.6. qRT-PCR Validation

First-strand cDNA was synthesized using the PrimeScript™ RT reagent Kit with gDNA Eraser (Perfect Real Time; Takara). The first-strand cDNA templates were diluted five-fold with EASY Dilution (for real-time PCR; Takara) and then used for a qRT-PCR assay, which was completed with SYBR^®^ Premix Ex Taq™ II (Tli RNaseH Plus; Takara) and the CFX connect Real-Time PCR Detection System (Bio-Rad, Hercules, CA, USA). The PCR program was as follows: 95 °C for 30 s; 40 cycles of 95 °C for 5 s and 60 °C for 30 s; 95 °C for 15 s, 60 °C for 1 min, and 95 °C for 15 s for the melting-curve analysis. The qRT-PCR solution consisted of 7.2 μL sterile distilled water, 0.4 μL 10 μM forward and reverse primers, 2 μL first-strand cDNA, and 10 μL SYBR^®^ Premix Ex Taq™ II (Tli RNaseH Plus; Takara). For each sample, a qRT-PCR was completed with three independent biological replicates and three technical replicates. The rose TCTP gene served as a reference gene [35]. Gene-specific primers (*RcAP1*, *RcLFY*, *RcSOC1*, *RcFUL*, -qPCR-F, and -qPCR-R) and reference primers (RTCTP-qPCR-F and RTCTP-qPCR-R) were designed for the qRT-PCR and the primers’ efficiency were tested (Supplementary Table S2). The relative expression data were analyzed according to the 2^−ΔΔCt^ method [53]. The results of the agarose gel electrophoresis and melting curve analysis were combined to verify the specificity of the PCR products.

### 4.7. Subcellular Localization of RcAP1

The pCAMBIA1302-GFP expression vector carrying the CaMV 35S promoter was digested with restriction enzymes *Nco*I and *Spe*I. The full-length *RcAP1* ORF (without a stop codon) amplified with primers *RcAP1*-GFP-F and *RcAP1*-GFP-R was ligated into the linearized pCAMBIA1302-GFP vector according to the homologous recombination method of the In-Fusion HD Cloning Kit (Takara). The subcellular localization of *RcAP1* was examined by transforming isolated maize protoplasts with the prepared vectors using an established protocol [54]. The transformed protoplasts were incubated for 16 h and then stained for 10 min in a phosphate-buffered saline solution supplemented with 0.1 mg/mL 4′, 6-diamino-2-phenylindole (DAPI; Sigma-Aldrich, USA). The RcAP1 protein was localized based on GFP fluorescence, and the nuclear was stained by blue-fluorescent DAPI, which were detected with a laser confocal microscope (Leica TCS SP8, Leica, Solms, Germany). The laser type used for GFP fluorescence was VIS (Solid state laser 20 mW: 488 nm), and the laser type used for DAPI fluorescence was UV (Diode, 50 mW: 405 nm). The appropriate excitation and emission wavelengths for GFP and DAPI were 488/507 nm and 358/416 nm, respectively. The tests of subcellular localization were repeated in *Nicotiana benthamiana* leaves. The recombinant plasmid pCAMBIA1302::*RcAP1*::GFP and pCAMBIA1302::GFP were transformed into *Agrobacterium tumefaciens* GV3101 competent cells by the freeze-thaw method, respectively. Transformed GV3101 were injected into the leaves of *Nicotiana benthamiana* for transient expression. After being cultured for 48 h under long-day conditions of 16 h light, the cells were observed with a laser confocal microscope (Leica TCS SP8, Germany) under 488 nm.

### 4.8. Arabidopsis Thaliana Transformation

The pCAMBIA1302 expression vector was digested with restriction enzymes *Nco*I and *BstE*II. The full-length *RcAP1* ORF amplified with primers *RcAP1*-OE-F and *RcAP1*-OE-R was ligated into the linearized pCAMBIA1302 vector using an In-Fusion HD Cloning Kit (Takara). A schematic diagram of the T-DNA region of the pCAMBIA1302-35S::*RcAP1* construct is provided in Figure S3. The recombinant plasmid pCAMBIA1302::*RcAP1* was inserted into *A. tumefaciens* GV3101 competent cells using a freeze-thaw method, after which a floral dip method was used to transform *A. thaliana* plants [55]. The full-length primers were used to identify transformed plants. All transgenic plants were analyzed by RT-PCR and qRT-PCR, with leaf cDNA as the template, until T3 generation plants were obtained. Additionally, we counted the rosette leaves and recorded the number of days to flowering in the transgenic and WT plants.

### 4.9. RcAP1 Knockdown in ‘Old Blush’ Plants by the VIGS Method

A previously described VIGS method [49] was used to silence *RcAP1* in ‘Old Blush’ plants. Primers pTRV2-*RcAP1*-F and pTRV2-*RcAP1*-R were used to obtain a 405-bp fragment of the *RcAP1* coding sequence. The fragment was cloned into the *BamH*I/*EcoR*I-cleaved TRV2 vector [56] with the In-Fusion HD Cloning Kit (Takara). The pTRV1, pTRV2, and pTRV2::*RcAP1* plasmids were separately inserted into *A. tumefaciens* AGL0 cells. The *A. tumefaciens* infiltration buffer containing 150 mM acetosyringone and the OD600 values was 1.0. The floral phenotypes were observed after each treatment (pTRV1 + pTRV2 and pTRV1 + pTRV2::*RcAP1*). Treatments were completed using 30 scions with young shoots approximately 2 cm long. Each scion has one axillary bud (stage S5), and one axillary bud on the grafted successful scion can produce one flower. The number of scions, which were treated by the *A. tumefaciens* infiltration buffer (pTRV1 + pTRV2::*RcAP1*), exhibited a delayed flowering phenotype. We randomly collected six shoot tips of the delayed flowering phenotype scions, about 2 cm from the top of shoots (pTRV1 + pTRV2::*RcAP1*), as well as six flower buds of control scions, which may already have been at S8 or S9 (pTRV1 + pTRV2). The mix samples were immediately frozen in liquid nitrogen and stored at −80 °C until used for a qRT-PCR validation. The VIGS tests were repeated three times. For each sample, the qRT-PCR was completed with three technical replicates.

## Supplementary Materials

Supplementary materials can be found at https://www.mdpi.com/1422-0067/20/14/3557/s1.

## Abbreviations

aa	Amino acids
VIGS	Virus-induced gene silencing
ORF	Open reading frame
NCBI	National Center for Biotechnology Information
qRT-PCR	Quantitative real-time polymerase chain reaction
WT	Wild-type
RACE	Rapid-amplification of cDNA ends
GFP	Green fluorescent protein
DAPI	4′,6-diamino-2-phenylindole
