# Supplementary material for: RcAP1, a Homolog of APETALA1, is Associated with Flower Bud Differentiation and Floral Organ Morphogenesis in Rosa chinensis
Source: Int J Mol Sci. 2019 Jul 20;20(14):3557. doi: 10.3390/ijms20143557 (PMC6679073; doi:10.3390/ijms20143557)
Supplement: Supplementary file 1 [file ijms-20-03557-s001.pdf]

International Journal of
Molecular Sciences

Supplementary Materials:

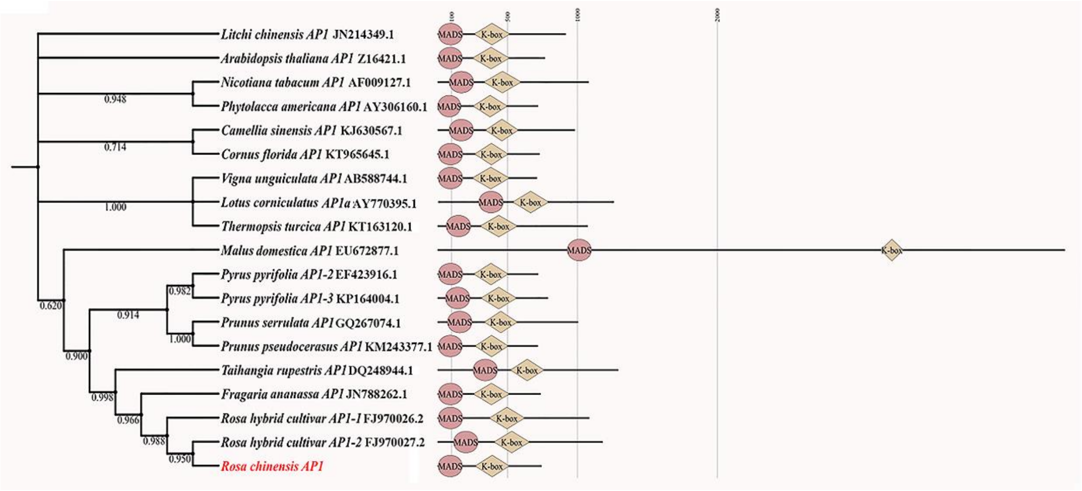

Figure 1. Phylogenetic analysis of *RcAP1* and *AP1* genes from other species based on the complete mRNA sequences. *RcAP1* investigated in this study is highlighted in red. The tree was constructed with MEGA 7.0 software using the neighbor-joining method. A total of 1,000 replicates were used for the bootstrap analysis. The bootstrap values (> 50%) are shown below the node. The nucleotide sequences of all *AP1* genes were obtained from the NCBI database. Accession numbers and simplified diagrams of conserved domains are provided after the species names. Dark red ovals represent the location of the MADS domain and light-yellow diamonds represent the location of the K-box domain. Straight lines represent the length of the gene.

The GenBank IDs of the *A. thaliana* type II MADS-box genes encoding the aa sequences used for the phylogenetic analyses were as follows: AEC10582.1 *AtAGL6*; NP_568929.1 *AtAGL8*; AAP37691.1 *AtAGL12*; AEE80158.1 *AtAGL13*; AEE83062.1 *AtAGL14*; AED91941.1 *AtAGL15*; ABN04784.1 *AtAGL16*; AEC07331.1 *AtAGL17*; ABF83664.1 *AtAGL18*; AEE84684.1 *AtAGL19*; NP_182090.1 *AtAGL20*; ABE77409.1 *AtAGL24*; AAO42844.1 *AtAGL27*; AED97992.1 *AtAGL31*; ACB88830.1 *AtAGL32*; BAH56782.1 *AtAGL42*; AAO64796.1 *AtAGL44*; AEE36053.1 *AtAGL66*; ABF19021.1 *AtAGL68*; AAS88775.1 *AtAGL67*; AED97997.1 *AtAGL69*; BAH56750.1 *AtAGL70*; AED96140.2 *AtAGL71*; AED96137.1 *AtAGL72*; AED97997.1 *AtAGL79*; AEE30200.1 *AtAGL104*; AAP12873.1 *AtSEP1*; AAW38979.1 *AtSEP2*; ACF75546.1 *AtSEP3*; AEC05740.1 *AtSEP4*; ABR46217.1 *AtFLC*; AED92817.1 *AtPI*; AEE79216.1 *AtAP3*; ACF09414.1 *AtSVP*; BAH30315.1 *AtCAL1*; AEE34887.1 *AtAP1*; AEE82819.2 *AtSTK*; AEE79831.1 *AtSHP1*; ABK59682.1 *AtSHP2*; and BAH30327.1 *AtGOA*.

The Gene IDs of the type II MADS-box genes from the *R. chinensis* 'Old Blush' genome database encoding the aa sequences used in phylogenetic analyses were as follows: Chr4g0429931 *RcAGL9*; Chr7g0229221 *RcAGL11*; Chr7g0230721 *RcAGL12*; Chr2g0109981 *RcAGL18*; Chr7g0199781 *RcAGL19*; Chr1g0318281 *RcMADS23*; Chr7g0205071 *RcAGL24*; Chr4g0439551 *RcAGL30*; Chr1g0376271 *RcAGL41*; Chr7g0240061 *RcAGL104*; Chr6g0269991 *RcANR1*; Chr1g0351671 *RcMADS6*; Chr7g0229221 *RcMADS27*; Chr6g0296731 *RcPMADS2*; Chr2g0111271 *RcSVP*; Chr5g0020391 *RcFBP24*; Chr7g0183521 *RcEJ2*; Chr4g0437851 *RcCMB*; and Chr2g0151031 *RcAG*.

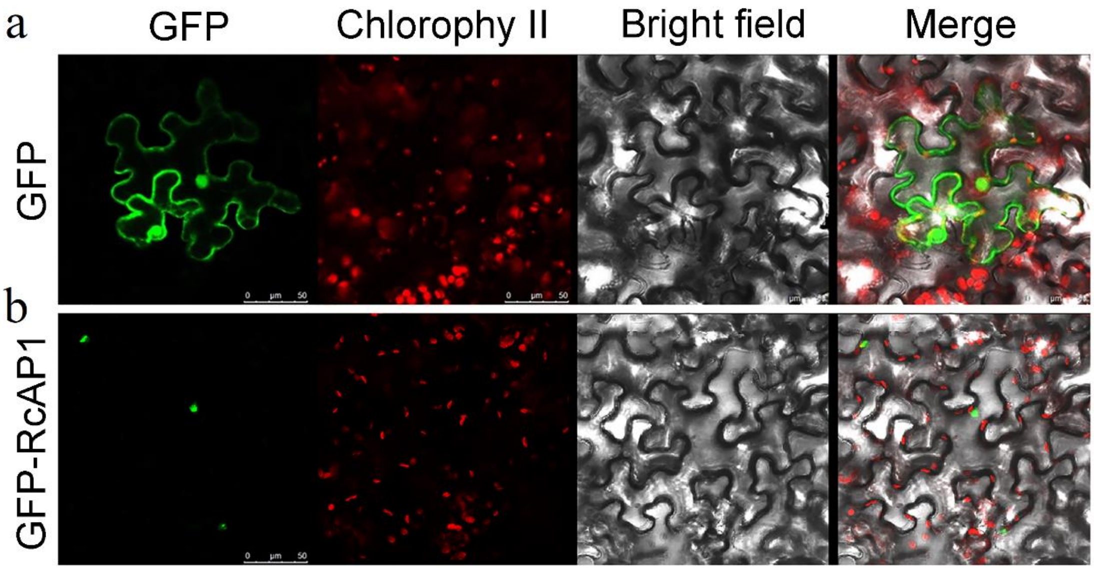

Figure 2. The subcellular localization of *RcAP1*. Driven by the CaMV 35S promoter, the GFP alone and GFP-*RcAP1* fusion protein were transiently expressed in Nicotiana benthamiana leaves. (a) The 35S::GFP subcellular localization figure . (b) The 35S::GFP::*RcAP1* subcellular localization figure. The cells were observed with a laser confocal microscope under 488nm blue light. Scale Bar, 50 μm.

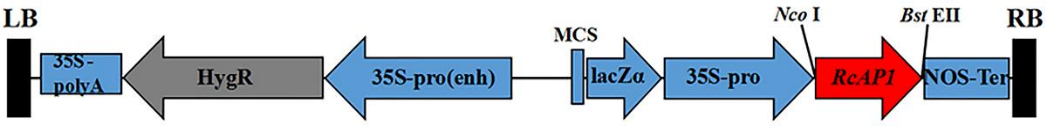

Figure 3. Schematic diagram of the T-DNA region of the pCAMBIA1302-35S::*RcAP1* construct. LB, left border of the region; 35S-polyA, CaMV 35S polyA; HygR, hygromycin(R); 35S-pro(enh), CaMV 35S promoter (enhanced); MCS, multiple cloning site; lacZα, lacZ alpha gene; 35S-pro, CaMV 35S promoter; *RcAP1*, ORF of *RcAP1* were inserted between the Nco Ⅰ and Bst EⅡ sites; NOS-Ter, nopaline synthase terminator; RB, right border of the region.

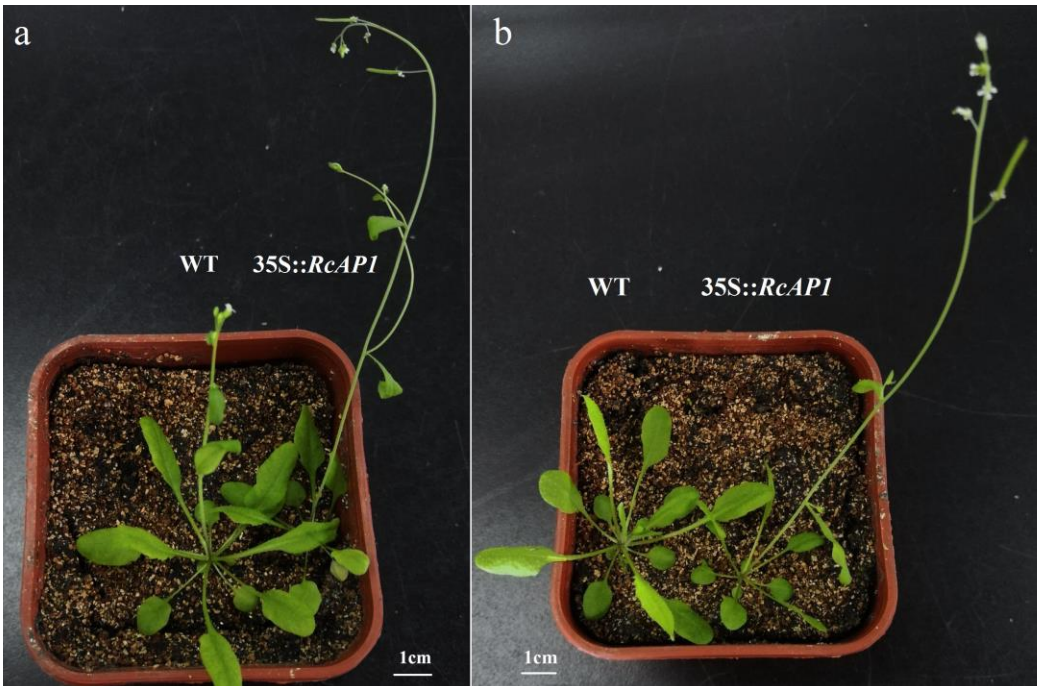

Figure S4. The phenotypes of other lines of 35S::*RcAP1* transgenic plants. (a) The phenotypes of Line 4. Bar, 1 cm. (b) The phenotypes of Line 5. Bar, 1 cm, WT, wild type of *A. thaliana*.

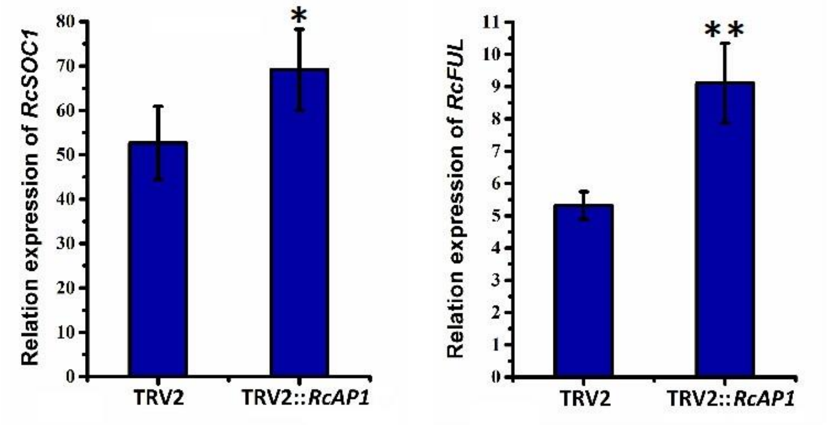

Figure 5. Quantitative analysis of *RcSOC1* and *RcFUL* expression in TRV2::*RcAP1* and TRV2 scions. Values are means + SD of three biological replicates. * and ** represent significant differences at $P < 0.05$ and $P < 0.01$, respectively (Dunnett's test).

Table 1. The cis-acting elements information of proRcAP1.

Site Name	The direction of the Strand	Position	Function of the site	Organism	Sequence
CCGTCC-Box	+	-429 to -423	meristem specific activation	*Arabidopsis thaliana*	CCGTCC
EM2	+	-1323 to -1314	floral meristem determinacy	*Arabidopsis thaliana*	CCTATTTAAG
RY	+	-1177 to -1169	flowering promoting	*Oryza sativa*	CATGCATG
DREB2A	-	-1079 to -1087	cold regulated	*Arabidopsis thaliana*	TGCCGACAA
HSE-1	-	-713 to -722	heat stress responsiveness	*Hordeum vulgare*	AAAAATTCTAC

Table 2. Primers used in this study. Tables should be placed in the main text near to the first time they are cited.

Primer name	Sequence (5'→3')
RcAP1-F	GGAAGGGGTAGGGTTCAGCT
RcAP1-R	GTTATTTTGCTCCTGCATTGC
3′ GSP	AAAGAAACCTTAGGCACTATTTGGGAGA
5′GSP	GATCGAATTTGCTTAAGAGAATTGTCAA
UPM	CTAATACGACTCACTATAGGGCAAGCAGTGGTATCAACGCAGAGT
RcAP1-full-F	ATGGGAAGGGGTAGGGTTCAG
RcAP1-full-R	TCATGAAGCAAAGCATCCAAG
RcAP1-qPCR-F	CATGAGTCTATGTCCGAACTTCA
RcAP1-qPCR-R	CCGCTACATTCTTCTCCTTCTC
RcLFY-qPCR-F	GCTATGGATGCTCTCTCTCAAG
RcLFY-qPCR-R	CTCTCCTAATCCTCCGAAGC
RcSOC1-qPCR-F	CAGATGAGGCGGATAGAGAAC
RcSOC1-qPCR-R	TCGGAGAGAAGATGATGAGC
RcFUL-qPCR-F	CTACATCACCACCAGCCCTA
RcFUL-qPCR-R	CATCTTCATCCATTGCGTCT
RTCTP-qPCR-F	GGGTGATGATGCAGCTTT
RTCTP-qPCR-R	TTAGCACTTGACCTCCTTCA
RcAP1-pro-SP1	GATTTGGAATCACAGGGTAACTGGAC
RcAP1-pro-SP2	GAGAATATACTTGAACGCTACGAGC
RcAP1-pro-SP3	TCTGTCTTGTGCGATGCTCAGGTTG
RcAP1-GFP-F	GGACTCTTGACCATGGATGGGAAGGGGTAGGGTTCAG
RcAP1-GFP-R	CTTCTCCTTTACTAGTTGAAGCAAAGCATCCAAGGT
RcAP1-OE-F	GGACTCTTGACCATGGATGGGAAGGGGTAGGGTTCAG
RcAP1-OE-R	ATTCGAGCTGGTCACCTCATGAAGCAAAGCATCCAAGGT
AtActin-F	ATGGTTGGTATGGGTCAG
AtActin-R	CCTCGTAGATTGGCACAGT

© 2018 by the authors. Submitted for possible open access publication under the terms and conditions of the Creative Commons Attribution (CC BY) license (http://creativecommons.org/licenses/by/4.0/).
